# Necrology

**Published:** 1899-07

**Authors:** 


### NECROLOGY.

James Milton Rose, V.S., died at Wilkinsburg, Pa., July 15, 1899, after a long illness. A practitioner for more than thirty years, most of which time was at Columbus, Ohio, though he traveled much in the performance of ridgling castration. A non-graduate, but registered in Ohio and Pennsylvania, in the latter State as a sojourner, he was in later years granted a certificate from the Ohio State Board of Examiners under a severe protest by Prof. H. J. Detmers, whose resignation from the Board followed as a result. The certificate and registration as a sojourner were a source of pending litigation in Pennsylvania, and which now closes by the death of Dr. Rose.

Dr. J. W. Ferguson, graduate of Ontario Veterinary College, Class of 1881, located at Bay City, Michigan, died in June, from injuries received from the kick of a horse he was treating. Dr. Ferguson was also a master horseshoer, and took a keen interest in the work of his craft at home and throughout the country.

